# Navigating the Frontiers of Machine Learning in Neurodegenerative Disease Therapeutics

**DOI:** 10.3390/ph17020158

**Published:** 2024-01-25

**Authors:** Yoonjeong Cha, Mohamedi N. Kagalwala, Jermaine Ross

**Affiliations:** Alleo Labs, San Francisco, CA 94105, USA; yj@alleolabs.com (Y.C.); mo@alleolabs.com (M.N.K.)

**Keywords:** machine learning, drug discovery, multiple sclerosis, amyotrophic lateral sclerosis, Parkinson’s disease, Alzheimer’s disease, Huntington’s disease

## Abstract

Recent advances in machine learning hold tremendous potential for enhancing the way we develop new medicines. Over the years, machine learning has been adopted in nearly all facets of drug discovery, including patient stratification, lead discovery, biomarker development, and clinical trial design. In this review, we will discuss the latest developments linking machine learning and CNS drug discovery. While machine learning has aided our understanding of chronic diseases like Alzheimer’s disease and Parkinson’s disease, only modest effective therapies currently exist. We highlight promising new efforts led by academia and emerging biotech companies to leverage machine learning for exploring new therapies. These approaches aim to not only accelerate drug development but to improve the detection and treatment of neurodegenerative diseases.

## 1. Introduction

The average investment in bringing a new drug to market ranges between $314 million and $2.8 billion, spanning over 10 to 15 years [1,2]. Despite rapid innovations in biotechnology equipment aimed at reducing the operating costs, drug development is primarily dependent on classical methods for assessing the safety and efficacy of drug candidates [3,4]. Such methods are associated with a number of pain points, including but not limited to patient stratification, target identification, high-throughput screening, drug design and optimization, biomarker discovery, and clinical trial design. Each exercise often employs an expensive brute-force approach that would largely be overlooked if not for the high attrition rates in drug development: oncology drugs have a 3.4% overall probability of success of gaining approval from the United States Food and Drug Administration (FDA); neuroscience drugs 15%; autoimmune/inflammation drugs, 15.1%; cardiovascular drugs, 25.5%; and vaccines 33.4% [5]. In this review, we will explore recent progress in machine learning (ML) that has enabled innovative approaches along the pipeline of CNS drug discovery. These promising methodologies seek to improve not only the cost and timelines of drug development but also the efficacy of drugs targeting neurodegenerative diseases.

## 2. Currently Approved Treatments for Neurodegeneration

The most common neurodegenerative diseases include Alzheimer’s disease (AD), Parkinson’s disease (PD), multiple sclerosis (MS), amyotrophic lateral sclerosis (ALS), and Huntington’s disease (HD) [6,7]. While significant progress has been made in understanding these disorders, there have been a limited number of effective treatments developed to modify the disease progression and severity in patients. For example, Alzheimer’s disease is the seventh leading cause of death in the United States and accounts for the greatest number of dementia cases worldwide [8]. In the early 1980s, a US-based workgroup at the National Institute of Neurological and Communicative Disorders and Stroke (currently known as the National Institute of Neurological Disorders and Stroke) established an universally accepted criteria for the diagnosis and staging of AD which triggered a modern age of research in the field [9]. Despite four decades of rigorous scientific effort, only seven treatments for AD have been approved by the FDA, with only two new treatments, Aducanumab and Lecanemab, since 2003. Five of the approved treatments, including galantamine (Razadyne), rivastigmine (Exelon), donepezil (Aricept), memantine (Namenda), and memantine/donepezil (Namzaric), are widely considered to only briefly and modestly improve AD symptoms, ultimately failing to prevent or slow disease progression [10]. Similarly, there are only seven approved drugs for ALS, including tofersen (Qalsody), sodium phenylbutyrate/taurursodiol (Relyvrio), edaravone (Radicava), riluzole (Rilutek, Tiglutik, Exservan), and dextromethorphan/quinidine (Nuedexta) [11]. Like the approved AD medications, most of these ALS therapies do not reverse or stop progression but instead relieve symptoms or delay progression in patients [12,13].

## 3. Link between Heterogeneity and Novel Disease Targets in Neurological Disorders

### 3.1. Genetic Heterogeneity

While the complexity of neurological disorders may partly explain the lack of success in drug development in this field, there is a growing amount of evidence supporting heterogeneity among patients with AD [14,15,16,17,18], ALS [19,20,21], and PD [22,23,24]. For sporadic forms of common neurological disorders, clinical diagnosis has been broadly applied, where patients present varying clinical features, including but not limited to disease onset and progression, symptomology, and clinical outcome. However, standardized criteria for neuropsychological assessment have proven often insufficient for differential diagnosis, and the lack of robust biomarkers has complicated diagnostic and prognostic work-up for neurological cases [25]. Genetic studies have provided clarity on the causative mutations in familial forms of Alzheimer’s disease, where characterized variants in the amyloid precursor protein (APP), presenilin 1 (PSEN1), and presenilin 2 (PSEN2) genes have been shown to be nearly but not fully penetrant [26], whereas familial Parkinson’s mutations in genes like leucine-rich repeat kinase 2 (LRRK2), glucocerebrosidase (GBA), Parkin (PRKN), and alpha-synuclein (SCNA) have been useful for determining PD risk, diagnosis, and disease progression [27]. Despite extensive knowledge of the genetic factors among risk carriers, the clinical heterogeneity among these cases is not completely understood [28].

### 3.2. Publicly Available Repositories for Deciphering the Heterogeneity within Neurodegeneration

An initial step toward deciphering the heterogeneity of neurodegenerative diseases may require stratifying patients into distinct cohorts based on biological data. Over the past decade, several comparative studies have expanded access to rich neurodegenerative datasets derived from medical imaging and biospecimen samples, including brain magnetic resonance imaging (MRI), positron emission tomography, postmortem brain and peripheral nerve tissue, cerebrospinal fluid, plasma, and electroencephalographs. Such publicly available repositories include the Alzheimer’s Disease Neuroimaging Initiative (ADNI) [29], the Alzheimer’s Disease Data Initiative (ADDI) [30], the Religious Orders Study and Rush Memory and Aging Project (ROS MAP) [31], the Accelerating Medicines Partnership Program for Alzheimer’s Disease project (AMP-AD) [32], the Parkinson’s Progression Markers Initiative (PPMI) [33], the Answer ALS project [34], and the Target ALS project [35], as well as others (Table 1). Arguably, these resources represent neurodegenerative-based counterparts to oncology-based data initiatives such as The Cancer Genome Atlas Program (commonly known as TCGA), which provides access to 2.5 petabytes of multi-omics data across 33 cancer types [36].

### 3.3. Computational Approaches to Stratifying Patients in Oncology

There are illustrative examples of employing transcriptomics and ML to subtype cancer patients based on biologically relevant associations, offering a starting point for applying similar approaches to classifying patients with neurodegenerative disorders. For example, a large body of literature has shed light on the genomic and epigenomic deregulation in cancer biology and its relationship to clinical heterogeneity. Breast cancer is widely known to be a highly heterogeneous disease, with differences observed across genomic, epigenomic, transcriptomic, and proteomic data [37]. Several bioinformatic approaches have been employed to unravel the patient stratification across different cancer patients like BRCA1/2 (breast cancer 1/2) mutation carriers. Notably, transcriptome analyses have revealed gene expression differences comparing BRCA1 and BRCA2 subjects as well as between breast and ovarian cancer patients [38]. Similarly, lung cancer studies have found considerable variation among histological samples associated with clinicopathological features [39], and gene expression analysis confirmed unique transcriptional profiles among lung adenocarcinoma and squamous cell carcinoma subtypes [40]. Further, classical ML methods, such as unsupervised learning, have demonstrated significant improvement in subclassifying tumors using gene expression data [40,41]. In particular, clinically relevant subtypes were characterized among luminal breast cancer samples by utilizing consensus clustering, an unsupervised ML technique that offers improvements in stability over the classical clustering methods [42,43,44].

### 3.4. Applications of ML to Stratifying Patients with Neurodegeneration

Beyond consensus clustering, more advanced ML algorithms have achieved comprehensive patient subtyping via the integration of diverse data types. Such examples encompass, but are not restricted to, Similarity Network Fusion (SNF), Pattern Fusion Analysis (PFA), NEMO (Neighborhood-Based Multi-Omics Clustering), non-negative matrix factorization (NMF), Subtype-GAN, and Perturbation Clustering for Data Integration and Disease Subtyping (PINS). While NEMO, SNF, and PINS are primarily based on similarity networks, PFA and NMF are grounded in the principles of dimensionality reduction. Recently, NMF was employed to stratify a large cohort of ALS patients based on samples generated from the Target ALS project [45]. Clustering analysis revealed three unique ALS subgroups, which were defined by transcriptional differences in biologically relevant mechanisms, including oxidative stress, reactive gliosis, and RNA dysregulation. ALS subtype patients associated with RNA dysregulation were linked to elevated levels of TAR DNA-binding protein 43 (TDP-43), a regulator of RNA processing known for its pathogenic role in ALS. Consistent with this notion, ALS subtype patients with RNA dysregulation exhibited initial limb symptoms, with prior research associating limb onset with TDP-43 pathology [46]. With an increasing body of evidence suggesting the involvement of RNA dysregulation in ALS [21], patient stratification presents an effective approach to discovering novel targets best suited for precision therapy.

## 4. Computational Approaches to Lead Discovery

### 4.1. Overview of ML in Lead Discovery

Lead discovery is an important stage in the drug discovery process. During this phase, chemical compounds, aimed against a specific target of interest, are identified and optimized to exert an ideal biological effect [47]. The latest research supports the potential of ML to improve the efficiency of pharmacological development. Concretely, drug hunters have applied ML to various bottlenecks of lead discovery, including hit-to-lead and lead optimization, and have developed approaches to the computational prediction of protein structures, virtual screening via structure-based/ligand-based methods, and the physicochemical optimization of lead drug candidates [47,48]. While generally agnostic to the disease area, the ML tools and methods described below have shown immense value in the field of CNS drug discovery.

### 4.2. Binding Site and Protein Structure Prediction

Computer-aided lead discovery starts with employing the available structural information on a disease target. Proteins are commonly studied as three-dimensional (or tertiary) structures, traditionally obtained using various prevalent methods such as X-ray crystallography, NMR spectroscopy, and cryo-electron microscopy [49]. The structural information is then pre-processed and analyzed to identify potential ligand-binding sites [50]. There are a number of existing algorithms available for binding pocket prediction (Table 2), including but not limited to Schrödinger’s SiteMap [51], Fpocket [52], DoGSiteScorer [53], and Q-SiteFinder [54]. The foundation of these tools varies widely, encompassing diverse technologies aimed at achieving accurate prediction. For example, SiteMap employs a grid-based method to evaluate the free energy profiles and geometry of the putative ligand motifs present on a protein target, whereas Fpocket is largely restricted to resolving binding cavities based on geometry alone. While these techniques are frequently used throughout CNS drug discovery [55,56,57], Fpocket and SiteMap utilize computational geometry and physics-based principles as opposed to ML. In contrast, recent advances have applied convolutional neural networks (CNNs) to resolving the putative functional pockets within neurodegenerative proteins. A CNN is a neural network that detects patterns in the input data, such as amino acids in proteins or atomic symbols in compounds. DeepSite is a deep CNN trained on over 7000 protein structures curated from a publicly available annotated database called sc-PDB [58], which comprises binding sites characterized from protein structures found in the Protein Data Bank [59]. Recently, DeepSite analysis revealed allosteric binding motifs in a neuronal protein known as Synapsin III (Syn III) and highlighted the structural interaction between Syn III and methylphenidate, a monoamine reuptake inhibitor used for treating attention deficit hyperactivity disorder [60]. Syn III is a member of the synapsin protein family, a group of evolutionarily conserved phospho-proteins crucial for regulating synaptic transmitter release and facilitating neuronal communication, and has been recently been associated with the aggregated α-synuclein found in PD and dementia with Lewy bodies (DLB) [61]. While relatively new compared to traditional prediction algorithms, deep CNNs have the potential to improve or complement geometry-based and physics-based predictions of the ligan-binding sites characterized in experimentally validated protein structures.

Due to the practical challenges associated with crystallography and NMR spectroscopy [62], there has been a growing trend in the accurate de novo prediction of protein structures using bioinformatics and ML (Table 2), including RoseTTAFold [63], I-TASSER [64], AlphaFold [65], and QUARK [66]. Similar to SiteMap, conventional predictions of protein structures rely on the principles governing protein energy functions—mathematical models that measure the energy linked to the shape or arrangement of a protein given a particular amino acid sequence. While energy-based modeling is computationally expensive, CPUs (Central Processing Units) and GPUs (Graphics Processing Units) have greatly improved over the years, which has yielded better predictions. In addition, parallelization and distributed computing have significantly increased the capacity to run simulations and computations at a large scale [67]. For example, NVIDIA’s CUDA (Compute Unified Device Architecture) has allowed developers to maximize the potential from GPUs for deep learning, including developing more effective force fields used in energy-based modeling [68].

AlphaFold and RoseTTAFold have become two widely adopted tools for modern protein structure prediction. Both deep learning tools can be used for ab initio folding, which is a method for predicting protein structures based solely on amino acid sequences. Conversely, template-based methods leverage existing experimental structure data to make de novo predictions. While RoseTTAFold combines both template-based modeling and ab initio folding, AlphaFold largely depends on ab initio folding, with lesser importance of the templates. Both AlphaFold and RoseTTAFold have been used to study the protein targets associated with neurodegeneration. PINK1 (PTEN-induced putative kinase 1) is a serine/threonine kinase known for its role in mitophagy and its impact on AD, ALS, HD, and PD [69]. Structure and mutagenesis studies have revealed disease-linked mutations within the functional kinase domain of PINK1, including at the 288th amino acid position—a serine residue (Ser288) crucial to autophosphorylation [70]. In contrast, there are several PINK1 mutations located in regions not included in the published structures of PINK1 [70]. AlphaFold analysis revealed the complete structure of human PINK1, including the presence of an alpha helix in the N-terminal region. Confirmed using mass spectrometry, the domain was subsequently shown to be necessary for Ser228 autophosphorylation and PINK1 activation, exhibiting a potential therapeutic mechanism in PINK1 patients [70]. Comparisons between AlphaFold and RoseTTAFold have also been conducted in structural prediction studies. Genome-wide association studies have revealed disease-associated mutations in PSEN1, APP, APOE (Apolipoprotein E), and TREM2 (Triggering Receptor Expressed on Myeloid Cells 2) [71], well-studied proteins that are the focus of therapeutic intervention for AD [72,73,74,75]. Protein structure predictions were carried out for all four proteins using both AlphaFold and RoseTTAFold to assess the accuracy of each modeling method against experimentally validated structures. The benchmark performance was evaluated using two metrics for structural similarity: Root Mean Square Deviation (RMSD) and Template Modeling score (TM-score). RMSD is frequently more effective in capturing the overall general structural similarity, even in instances where no experimental structure reference is available (or ab initio predictions) [76]. The TM-score is arguably more robust than RMSD as it considers the entire structural alignment, enabling it to detect finer structural variations, although this metric is more applicable to template-based predictions [76]. The TM-scores and RMSD estimates revealed a high degree of similarity between AlphaFold and RoseTTAFold when predicting the protein structures of PSEN1, APP, APOE, and TREM2 [77]. The modeling predictions also resolved gaps in PSEN1 that were not captured using X-ray crystallography or cryo-electron microscopy [77]. Generally, X-ray crystallography is not very sensitive to the mobility of proteins, whereas template-based predictions may be a powerful tool for elucidating the intrinsically flexible segments of partially captured proteins.

### 4.3. Hit Identification via Virtual Screening

During the hit identification stage of drug discovery, a proven method for identifying chemical hits to targets includes purifying the disease target proteins, establishing robust biochemical assays, conducting high-throughput screening (HTS) of chemical libraries, and separating out active hits [78]. Although automation and miniaturization have contributed to reducing costs, the well-established practice remains relatively expensive and inefficient, particularly for novel and higher-risk targets, as the costs are tied to the size of the chemical library and its scope [79]. To mitigate risks, there has been a greater focus on utilizing ML for binding prediction between targets and ligands. In broad terms, there are two approaches to virtual HTS: ligand-based screening and structure-based screening. Structure-based screening can be further divided into complex-based and pair-based models [80]. While ligand-based screening typically relies on similarity measures, the majority of structure-based models integrate some application of ML. For structure-based screening, quantitative structure–activity relationships (SARs) can be predicted with or without employing a tertiary structure, which, until recently, was largely confined to experimentally resolved structures, but now, with advancements like the AlphaFold and RoseTTAFold algorithms, has seen expanded possibilities. Instead of tertiary structures, pair-based screening involves training models using primary representations of proteins in the form of SMILES as input, coupled with biochemical activity data, which benefit from being more deployable compared to complex-based screening [81]. Specifically, training billions of compounds using deep CNNs based on SMILES representation is significantly less computationally expensive relative to physics-based 3D docking methods.

Recently, virtual ligand-based HTS was utilized to discover inhibitors targeting the α-synuclein protein [56]. α-synuclein is a pathological hallmark of PD and DLB, and its aggregation is associated with the degeneration of the dopaminergic neurons residing in the substantia nigra pars compacta, a brain region involved in motor planning [56]. To identify α-synuclein binders, a virtual HTS was performed using SwissSimilarity (Table 2), a web-accessible tool for identifying putative hits using a diverse collection of promising and validated small molecule libraries including but not limited to 3071 approved drugs and 2989 drug candidates from the ChEMBL 29 database, over 320,000 commercially available molecules from the SPECS library, over 9 million molecules from the ZINC20 database, and over 30 million molecules from the Enamine “REAL” catalog [82]. To prime the search, SwissSimilarity analysis was conducted using two known α-synuclein binders, namely SynuClean-D and ZPD-2. Both α-synuclein hits represent tool compounds validated in cell-based experiments but lack drug-like properties [83]. Ligand-based screening using the SPECS library revealed analogs of SynuClean-D and ZPD-2, which were selected by leveraging multiple molecular fingerprinting methods. Each of these approaches facilitate similarity analysis by offering a distinct representation of the molecular structures, such as electrostatic properties, predefined chemical substructures, and the distribution of atomic charge [84]. The SwissSimilarity analysis identified 363 putative analogs of SynuClean-D and ZPD-2, which were further filtered for ideal drug-like properties, the absence of PAINS (pan-assay interference compounds), and commercial availability. A final set of 34 structures was selected for experimental validation, including a structurally similar analog denoted as MeSC-04. Cell-based assays showed that MeSC-04 is a potent inhibitor of α-synuclein amyloid formation. Fpocket and SiteMap were employed to identify the binding pockets of α-synuclein, and molecular docking was performed to evaluate the binding interaction between MeSC-04 and the identified motifs. The molecular docking studies demonstrated binding interactions consistent with the previously reported findings involving SynuClean-D and α-synuclein, supporting the utility of ligand-based screening for chemical hits [56].

Pair-based screening is focused on predicting the quantitative SAR in protein–protein or protein–ligand interactions independent of knowing the native structure of the proteins or the ligands [80]. Ligands are inputted as SMILES, molecular fingerprints, or molecular graphs, whereas proteins are represented using full or partial sequences of amino acids. Pair-based screening can be performed using random forests, support vector machines, multilayer perceptrons, and neural networks [80]. Advanced architectures commonly utilize different types of neural networks, specifically recurrent neural networks, deep CNNs, and graph CNNs. Several CNN-based applications, such as DeepDTA and GraphDTA, are open-source and available for performing pair-based screening of ligand libraries (Table 2). For example, DeepDTA was recently utilized to identify hits for Mitofusin-2 (Mfn2), a GTPase associated with mitochondrial dysfunction that is implicated in the underlying pathology of AD [85]. Mfn2 is one of two paralogs of the mitofusin protein family, which are primarily responsible for the fusion of mitochondrial outer membranes [86]. DeepDTA was trained on a protein–ligand binding affinity database that consists of 1063 approved drugs called the Davis dataset, and its performance was compared to other models (Table 2), including GraphDTA (a graph CNN), DeepGS (a deep CNN), and a novel architecture called a three-tunnel deep neural network (a deep CNN denoted as 3-Tunnel DNN). To improve the training on the protein–drug binding affinity, a 3-Tunnel DNN distinguishes itself from other deep CNN models by explicitly integrating information from both positive samples (indicating protein–drug interactions) and negative samples (representing the absence of interactions), as well as incorporating protein sequences. When assessing the training performance, all tested models demonstrated comparable benchmarks, evaluated using metrics such as mean square error and the consistency index. Analysis of the 3-Tunnel DNN model screening revealed several approved drugs that exhibit potential to be repurposed for the inhibition of Mfn2 activity. Notably, Lamotrigine, Bosentan, Fluphenazine, Nabumetone, and Carbamazepine, featured in the leading drug hit list, are all medications previously investigated for their potential in AD treatment [85].

Complex-based screening aims to predict quantitative SARs in protein–protein or protein–ligand interactions by utilizing structural information on both the proteins and ligands [80]. Similar to pair-based models, complex-based methods consist of classical and modern ML approaches, commonly incorporating complex neural networks and encoding proteins as 3D grids [80,87]. For example, recent applications have employed deep CNNs for the structure-based screening of ligands against neurodegenerative protein targets, such as AMPA (α-amino-3-hydroxy-5-methyl-4-isoxazolepropionic acid) receptors. AMPA receptors are widely expressed in the CNS, and their dysfunction likely mediates the glutamate excitotoxicity underlying neuronal death and disease progression in MS [88]. A recent study utilized a deep-CNN-guided approach to identifying hits that may bind to an allosteric pocket located on one of the four subunits of AMPA receptors, known as glutamate receptor 2 (GluA2) [89], notable for its role in the regulation of Ca^2+^ permeation and voltage rectification [90]. Complex-based screening was conducted utilizing Atomwise’s proprietary CNN (AtomNet) for predict the binding affinity of small molecules to GluA2 (Table 2) [90]. The effects of 50 putative GluA2 hits were validated using a cell-based assay to assess the glutamate-mediated excitotoxicity. In vitro models confirmed that glutamate-mediated excitotoxicity was inhibited by several hits, including highly potent compounds denoted as YH668, ZCAN155, and ZCAN262 [90]. Pharmacokinetic studies revealed that ZCAN262 also had good oral bioavailability and brain exposure. Animal studies demonstrated that ZCAN262 treatment is sufficient to rescue myelination and axon integrity in EAE mice, an MS mouse model [90].

### 4.4. Lead Optimization Using ML

After hit identification, drug discovery teams have often embarked on intensive campaigns of medicinal chemistry to characterize drug candidates for Investigational New Drug (IND)-enabling studies. Such efforts of drug discovery can be broken down into the following stages: hit-to-lead, lead identification, and lead optimization. All phases of development involve rapid analog generation to improve their physicochemical properties and advance potential leads toward having drug-like characteristics. Collectively, the objective is to satisfy a set of predefined requirements known as a Target Product Profile (TPP) [91,92]. While context-dependent, the TPP broadly consists of thresholds for safety and efficacy. Concretely, the focus is on optimizing the parameters for ADME (absorption, distribution, metabolism, and excretion) to improve the overall bioavailability and target engagement while also attempting to reduce any safety/toxicity liabilities. Examples of ADME properties include aqueous solubility, membrane permeability, microsomal stability, and blood–brain barrier (BBB) penetrance, whereas early safety and toxicity asssessment evaluates the inhibition of hERG (the human Ether-à-go-go gene) and CYP (cytochrome P450) activity [92].

To accelerate the discovery of CNS drug candidates, ML approaches have been developed to predict the optimization of ADME and toxicity. For example, DeePred-BBB is a deep CNN for predicting BBB permeability [93]. DeePred-BBB was trained on a broad set of 3605 compounds screened for BBB permeability and was benchmarked using the area under the curve (AUC). Compared to other published BBB permeability prediction models, DeePred-BBB performed relatively well with an AUC of 0.992. In contrast, the best reported AUC from another model is 0.98, which also employed a deep learning approach but was trained on a relatively smaller BBB dataset (462 compounds) [93]. Beyond DeePred-BBB, there are other emerging ML solutions for lead optimization, including those that incorporate generative artificial intelligence (AI) tools like large language models (LLMs), i.e., Bidirectional Encoder Representations from Transformers (BERT). For example, Mol-BERT was trained on datasets to predict not only BBB permeability but also clinical toxicity [94]. Applications like DeePred-BBB and Mol-BERT represent a promising new era of ML-guided drug design.

## 5. Industry Case Studies

**Figure 1:**
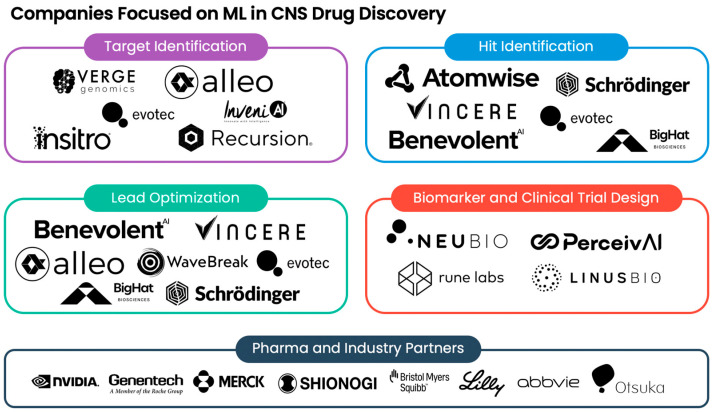
Selected companies using machine learning in CNS drug discovery. Illustrated is a summary of the biotech companies leveraging machine learning (ML) across different domains, including target identification, hit identification, lead optimization, biomarker design, and clinical trial design. Partner pharmaceutical and industry companies are also shown.

Over the years, several biotech companies have emerged with a focus on using cutting-edge ML approaches for CNS drug discovery (Figure 1). From target identification to clinical trial design, these biotech companies have leveraged ML to accelerate therapeutic discovery, rapidly establishing drug pipeline programs and state-of-the-art platform technologies. For example, several companies, such as Verge Genomics [95], Alleo Labs [96], Insitro [97], Evotec [98], InveniAI [99], and Recursion [100], have pioneered the development of ML platforms for CNS target identification. Meanwhile, Schrödinger, an industry leader in complex-based screening, recently partnered with Otsuka Pharmaceutical and Bristol Myers Squibb to perform hit identification and lead optimization for potential CNS therapies [101]. Similar to WaveBreak Therapeutics [102] and BenevolentAI [103,104], Vincere Biosciences is applying GPU-powered ML to lead discovery using its own proprietary software for screening and optimizing small molecules [105]. Currently, Vincere is actively pursuing inhibitors for USP30, a deubiquitinating (DUB) enzyme implicated in PD. Alleo Labs, a biotech developing ML-guided precision medicine, is employing LLMs for small-molecule optimization of lead inhibitors for novel AD and PD targets, including DUB enzymes [106]. AbbVie and BigHat Biosciences recently formed a collaboration to leverage BigHat’s ML design platform for treatments in neuroscience [107]. BigHat’s platform employs the principles underlying generative AI to characterize and optimize antibodies [108]. Verge Genomics has utilized its end-to-end ML technology to identify novel ALS targets and develop small-molecule therapeutics, namely VRG50635, an inhibitor of kinase PIKfyve (also known as Phosphoinositide Kinase, FYVE-Type Zinc-Finger-Containing). Verge successfully evaluated VRG50635 for its safety and tolerability in phase 1 clinical trials [109]. Beyond therapeutics, several biotech companies, such as NeuBio [110], Perceiv AI [111], Rune Labs [112], and LinusBio [113], are focusing on identifying robust biomarkers, as well as optimizing clinical trial design. Concretely, NeuBio is seeking to develop a blood test that can accurately diagnose disease in the earliest stages of development of neurodegeneration, by analyzing publicly available transcriptomic datasets from case–control studies of prodromal and early-stage disease using an evolutionary ML platform. NeuBio has assembled a panel of 141 RNA-based biomarkers that can be used for accurate diagnosis of AD, PD, and ALS. To inform patient selection and stratification when designing clinical trials for AD, Perceiv AI has developed a predictive ML platform that integrates different data types, such as fluid, genetic, and imaging biomarkers. In addition to advancing the field of artificial intelligence, NVIDIA has played a pivotal role in supporting ML-based biotech startups, such as Alleo Labs, Vincere, and Perceiv AI, through the NVIDIA Inception Program [114].

## 6. Conclusions

In this review, we summarized the advanced ML tools and approaches employed at various stages of CNS drug discovery. Given that patient stratification may be required to investigate new targets and treatments for neurodegeneration, we noted the utility of leveraging modern clustering algorithms to subtype patients using biological data, including the resources available via existing online repositories. We also examined several examples of employing more sophisticated neural networks to identify and design treatments during lead discovery. Lastly, we illustrated ongoing efforts to utilize ML for improving the clinical study design in neurodegenerative diseases. As these tools evolve, ML shows significant potential in reshaping the field of CNS drug discovery.

## Figures and Tables

**Table 1:** Publicly available repositories with neurodegenerative disease data.

Data Repository	Disease Area	Data Types	Reference
Alzheimer’s Disease Neuroimaging Initiative	AD	Brain magnetic resonance imaging, positron emission tomography, multi-omics, clinical, fluid biomarkers	[29]
Alzheimer’s Disease Data Initiative	AD	Multi-omics, clinical trial readouts	[30]
Religious Orders Study and Rush Memory and Aging Project	AD	Multi-omics, brain magnetic resonance imaging, neuropathology, clinical, fluid biomarkers	[31]
Accelerating Medicines Partnership Program for Alzheimer’s Disease	AD, PD, other NDDs	Multi-omics, brain magnetic resonance imaging electrophysiology	[32]
Parkinson’s Progression Markers Initiative	PD	Multi-omics, brain magnetic resonance imaging, clinical	[33]
Answer ALS Project	ALS	Multi-omics, clinical	[34]
Target ALS Project	ALS	Multi-omics, clinical	[35]

Alzheimer’s, AD; amyotrophic lateral sclerosis, ALS; Parkinson’s, PD; neurodegeneration, NDD.

**Table 2:** Selected computational tools available for predicting structure–activity relationships during CNS drug development.

Drug Discovery Application	Algorithm Examples	CNS Target Examples
Protein binding site prediction	SiteMap, Fpocket, DoGSiteScorer, Q-SiteFinder, DeepSite	Synapsin III
Protein structure prediction	RoseTTAFold, I-TASSER, AlphaFold, QUARK	PINK1, PSEN1, APP, APOE, TREM2
Ligand-based virtual screening	SwissSimilarity	α-synuclein
Structure-based virtual screening	DeepDTA, GraphDTA, DeepGS, 3-Tunnel DNN, AtomNet	Mfn2, GluA2

## Data Availability

Not applicable.
